# MicroRNA-328 Inhibits Renal Tubular Cell Epithelial-to-Mesenchymal Transition by Targeting the CD44 in Pressure-Induced Renal Fibrosis

**DOI:** 10.1371/journal.pone.0099802

**Published:** 2014-06-11

**Authors:** Cheng-Hsien Chen, Chung-Yi Cheng, Yen-Cheng Chen, Yuh-Mou Sue, Chung-Te Liu, Tzu-Hurng Cheng, Yung-Ho Hsu, Tso-Hsiao Chen

**Affiliations:** 1 Division of Nephrology, Department of Internal Medicine, Wan Fang Hospital, Taipei Medical University, Taipei, Taiwan; 2 Division of Nephrology, Department of Internal Medicine, Shuang Ho Hospital, Taipei Medical University, New Taipei City, Taiwan; 3 Department of Biological Science and Technology, College of Life Sciences, China Medical University, Taichung, Taiwan; VCU, United States of America

## Abstract

Epithelial-mesenchymal transition (EMT) occurs in stressed tubular epithelial cells, contributing to renal fibrosis. Initial mechanisms promoting EMT are unknown. Pressure force is an important mechanism contributing to the induction and progression of renal fibrogenesis in ureteric obstruction. In our study of cultured rat renal tubular cells (NRK-52E) under 60 mmHg of pressure, we found that the epithelial marker E-cadherin decreased and mesenchymal markers, e.g., α-smooth muscle actin, fibronectin and Snail, increased. Pressure also induced the expression of connective tissue growth factor and transforming growth factor-β. MicroRNA array assays showed that pressure reduced miR-328 at the initial stage of pressurization. We identified a potential target sequence of miR-328 in rat CD44 3′-untranslated regions. In contrast with the miR-328 expression, CD44 expression was up-regulated at the initial pressurization stage. We also found that miR-328 expression decreased and CD44 increased in ureteric obstruction kidneys in the animal study. CD44 siRNA transfection significantly increased E-cadherin expression and inhibited pressure-induced EMT. Both hyaluronan binding peptide pep-1 and osteopontin neutralizing antibody inhibited pressure-induced EMT. Our results suggest that miR-328-mediated CD44 transient upregulation is an important trigger of the pressure-induced EMT in renal fibrosis.

## Introduction

In the last decade, many studies used unilateral ureteric obstruction (UUO) in rodents as a model of renal fibrosis since the fibrotic response is compatible with human disease [1]. In UUO animal models, pressure in the obstructed ureter is up to 35–60 mmHg [2]. Sustained obstruction can convey pressure on the renal tubular system resulting in dilation of the collecting system, parenchymal thinning, tubular atrophy, and fibrosis [3]. An in vitro system was developed to study the influence of pressure on renal epithelial cells [4], [5]. Those studies revealed that pressure increases inducible nitric oxide synthase, transforming growth factor (TGF)-β, connective tissue growth factor (CTGF), and fibronectin. TGF-β and CTGF are necessary mediators for fibrogenesis [6]. Fibronectin is one of the extracellular matrix (ECM) components that accumulate during renal fibrosis [7], [8]. These results support that pressure force is an important cause contributing to the induction and progression of renal fibrosis in ureteric obstruction, but the mechanism of pressure-induced renal fibrosis, especially at the early stage, is still unknown.

Epithelial-to-mesenchymal transition (EMT), a major contributor to the pathogenesis of renal fibrosis, leads fully differentiated epithelial cells to change into matrix-producing myofibroblasts [9]. It is characterized by the loss of epithelial characteristics (E-cadherin) and increased mesenchymal phenotype (alpha smooth muscle actin [α-SMA], Snail, and fibronectin) [10]. Tubular epithelial cells obtaining a mesenchymal phenotype can enhance migratory capacity to transit from the renal tubular microenvironment into the interstitial space and maintain structural completeness by secreting ECM [11]. EMT is thought to be driven by extracellular stimuli, such as TGF-β, a well-established inducer [12]. Understanding the process of EMT is necessary for developing therapeutic strategies for progressive renal failure.

MicroRNAs (miRNAs) have been suggested to be involved in EMT of tubular epithelial cells. MicroRNAs are non-coding, single-stranded RNA molecules 19–25 nucleotides long, which can downregulate gene expression at the post-transcriptional level by pairing with the complementary sequences of the 3′-untranslated regions (3′UTR) of messenger RNA [13]. Evidence shows miRNA involvement in embryonic development, tumorigenesis, metastasis, metabolism, and many other physiological and pathological processes [14]. Studies found that miR-141, miR-200b and miR-205 can prevent TGF-β-induced EMT by downregulating ZEB1 and ZEB2, the two major transcriptional repressors of E-cadherin [15], [16]. MiR-200a can repress TGF-β2 expression to prevent renal fibrogenesis [17]. In vitro, miR-192 is induced by TGF-β1 and mediates TGF-β–induced collagen expression in mesangial cells [18]. Increased expression of miR-377 is also thought to regulate the expression of fibronectin [19]. These results suggest that some miRNAs play important roles in renal fibrosis and tubular epithelial cell EMT.

Our study investigated pressure-induced EMT and its early-stage mechanisms. MiR-328 has been reported to regulate zonation morphogenesis by targeting CD44 expression in human cells [20]. CD44, a widely distributed cell surface glycoprotein, mediates cell adhesion and migration in a variety of pathophysiological processes, including tumor metastasis, wound healing, and inflammation [21]. Hyaluronan (HA) and osteopontin (OPN) are the main ligands for CD44 [22], [23]. In rat renal tubular (NRK-52E) cells, we evaluated the miR-328/CD44 signaling involvement in pressure-induced EMT.

## Materials and Methods

### Ethics Statement

All animal experiments were approved by the Taipei Medical University Committee of Experimental Animal Care and Use.

### Reagents

Dulbecco’s modified Eagle’s medium (DMEM), fetal calf serum, and tissue culture reagents were from Life Technologies (Grand Island, NY, USA). All other chemicals of reagent grade were obtained from Sigma (St. Louis, MO, USA). Pep-1 (GAHWQFNALTVR) and scrambled control peptide (SATPASAPYPLA) [24] were synthesized by Kelowna International Scientific (Taipei, Taiwan). BSA antibody was purchased from Bioss (Woburn, MA, USA), and osteopontin neutralizing antibody from R&D Systems (Minneapolis, MN, USA).

### Cell Culture

Rat renal tubular cells (NRK-52E), purchased from the Food Industry Research and Development Institute (Hsinchu, Taiwan), were cultured in DMEM supplemented with an antibiotic/antifungal solution and 17% fetal bovine serum and were grown until the monolayer became confluent. The medium for the cultured cells was then changed to serum-free medium, and cells were incubated overnight before the experiment. To study the effects of pressure on cells in vitro, we established a novel pressure apparatus, as previously reported [4]. We applied 60 mmHg of pressure in this study considering the pressure in the obstructed ureter in animal models [2], [25]. The entire system was placed in a CO_2_ incubator to maintain a constant temperature (37°C) and humidity (95%).

### miRNA Microarray and Quantitative RT-PCR

Monolayers of NRK-52E cells were harvested, and total RNA was extracted with TRIzol reagent (Life Technologies, Grand Island, NY, USA) according to the manufacturer’s instructions. The isolated total RNAs were then analyzed by Welgene Biotech (Taipei, Taiwan) with Agilent miRNA microarray (Sanger miRBase v.14). For quantitative RT-PCR of miR-328, miRNAs in total RNA were reverse transcribed using TaqMan MicroRNA Reverse Transcription Kit (Life Technologies, Grand Island, NY, USA), and then applied in TaqMan MicroRNA Assays with specific primers of rat miR-328 (cat. no. 4427975, miRBase ID: rno-miR-328a-3p) according to the manufacturer’s instructions (Life Technologies). Reactions were run on a real-time PCR system (ABI PRISMH 7700, Applied Biosystems, Austin, TX, USA). Specific primers for U6 were used to normalize the amount of sample added. Relative amounts of the miR-328 were quantitated using the comparative Ct method. All quantifications were performed on triplicate samples of three separate experiments.

### miRNA and miRNA Inhibitor Transfection

Transfection of mature microRNAs (cat. no. 4427975) or inhibitors of rat miR-328 (cat. no. 4464066, Ambion Inc. Austin, TX, USA) was performed using Lipofectamine 2000 (Life Technologies) according to the manufacturer’s protocol. Transfection complexes were added to the cells at a final oligonucleotide concentration of 20 nM, and the medium was replaced 24 h later.

### Plasmid Constructs and 3′UTR Target Assay

For luciferase reporter experiments, the 3′-UTR of the CD44 gene was amplified by PCR from rat genomic DNA and inserted into the miR-Selection Fire-Ctx lentivector (System Biosciences, Mountain View, CA, USA) using the BamHI and EcoRI sites. The following primer sets were used to generate specific fragments: 5CD44BamHI: 5′-GGATCC TGCCTATGCCACTAACTTGAAA-3′; 3CD44EcoRIL: 5′-GAATTC AACCTGCTCATCTGGTGGTG-3′; 3CD44EcoRIS: 5′-GAATTC TCAGAAGTCCCCTCCACACT-3′. All inserts were confirmed by sequencing. Lentivector transfections were performed with LentiStarter kit (System Biosciences) according to the manufacturer’s instructions. The stable clones of control lentivector and the contructs CD44L and CD44S were applied in 3′UTR target analysis. Luciferase activity was measured after pressurization using the Luciferase Assay System (Promega, Madison, WI, USA). Three independent experiments were performed in triplicate.

### short interfering (si)RNA Transfection of CD44

We purchased rat CD44 siRNA (cat. no. 4390771) from Ambion Inc. Cells were grown to 70% confluence, and the siRNAs were transfected using the Lipofectamine 2000 transfection reagent (Life Technologies) according to the manufacturer’s instructions. We replaced the culture medium after 24 h for pressurization and the Western blot assays.

### Western Blot Analysis

Fifteen micrograms of NRK-52E lysate proteins were applied to each lane and analyzed by Western blotting. Antibodies of CTGF, Snail and fibronectin, purchased from Santa Cruz Biotechnology (Dallas, TX, USA), were diluted to 1∶200 for the assay. Antibodies of CD44 and TGF-β, purchased from Cell Signaling Technology (Danvers, MA, USA), were diluted to 1∶1000 for the assay. Antibodies of E-cadherin and α-SMA, purchased from Bioss, were diluted to 1∶1000 for the assay. The antibody of osteopontin was purchased from R&D Systems, and diluted to 1∶1000 for the assay. Peroxidase-conjugated anti-rabbit or anti-mouse immunoglobulin G (IgG; at a 1∶2000 dilution) was used as the second antibody for enhanced chemiluminescence (Thermo Scientific, Rockford, IL, USA). Data of protein bands on Western blots were also quantitated with QuantiScan software (Biosoft, Cambridge, UK).

### Induction of UUO in Rats

Male Sprague-Dawley rats weighing 200 to 220 g were obtained from BioLasco Taiwan (Taipei, Taiwan). Rats were unilaterally obstructed by ligation of the left proximal ureter for 7 days. Control rats were treated in the same way except that no ligature was made. The rats were housed in a central facility, subjected to a 12-h light-dark cycle, and provided with regular rat chow and tap water. They were anesthetized and sacrificed on day 7 after operation. The kidneys were harvested by laparotomy and fixed with formalin for immunohistochemistry.

### Immunohistochemistry

Sections 3–5 mm thick of formalin-fixed paraffin-embedded kidney specimens were made for IHC. Slides were deparaffinized in xylene and rehydrated with 100% ethanol, 95% ethanol and water serially. We immersed the slides into antigen-retrieval buffer (10 mM Tri-sodium citrate dihydrate, 0.05% Tween-20, pH 6.0) boiled at via water bath for 30 minutes. Slides were stained using an UltraVision Quanto HRP Detection kit (Thermo Scientific, Rockford, IL, USA) with specific antibodies according to the manufacturer’s instructions.

### Statistical Analysis

Data are presented as mean±standard deviation (s.d.). Between-group differences were determined using Student’s *t*-test, and *P* values of <0.05 were considered significant.

## Results

### Pressure-induced EMT

**Figure 1:**
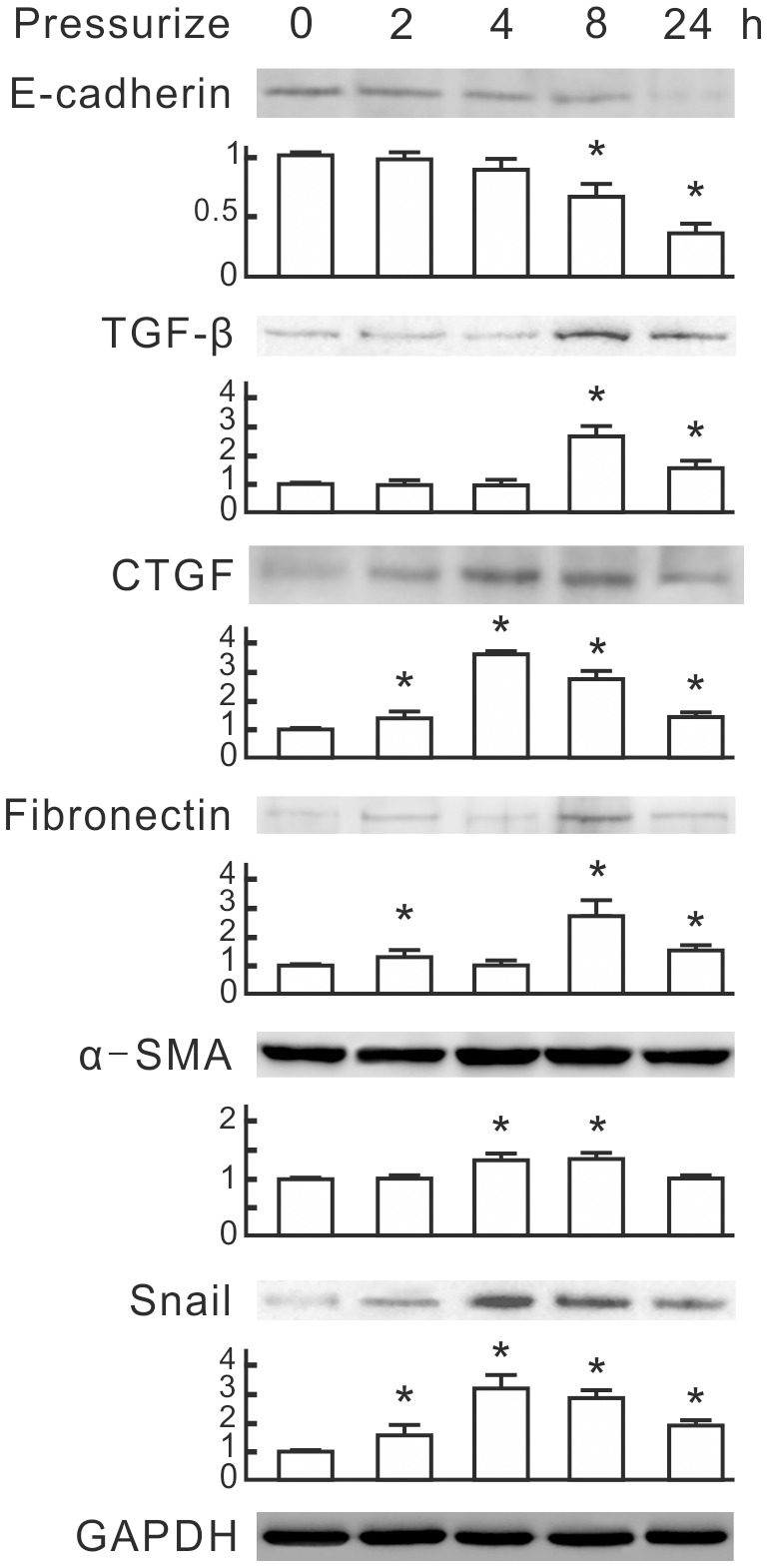
Expression of epithelial-mesenchymal transition (EMT) markers induced by a pressure of 60 mmHg in NRK-52E cells. NRK-52E cells were subjected to 60 mmHg of pressure for the indicated periods. Cellular E-cadherin, TGF-β, CTGF, fibronectin, α-SMA and Snail were detected by Western blotting. GAPDH was detected as the loading control. Relative increases in the protein bands are also presented in a bar chart form. Results are expressed as the mean±s.d. (*n* = 3). *P<0.05 vs. the group at 0 h. TGF-β, transforming growth factor-β; CTGF, connective tissue growth factor; α-SMA, alpha smooth muscle actin; GAPDH, glyceraldehyde-3-phosphate dehydrogenase.

To examine whether pressure induces EMT in NRK-52E cells, we monitored expression patterns of TGF-β and EMT markers. Western blot analysis revealed that pressure at 60 mmHg significantly reduced the epithelial marker E-cadherin at 8 and 24 h and induced TGF-β and fibronectin at 2, 8 and 24 h (Fig. 1). The pressure induced CTGF and Snail at 2h, with peaks at 4 h. The mesenchymal marker α-SMA was significantly induced at 4 and 8 h. These results reveal that 60 mmHg pressure induces EMT in NRK-52E cells.

### miRNA Expression under Pressure

**Figure 2:**
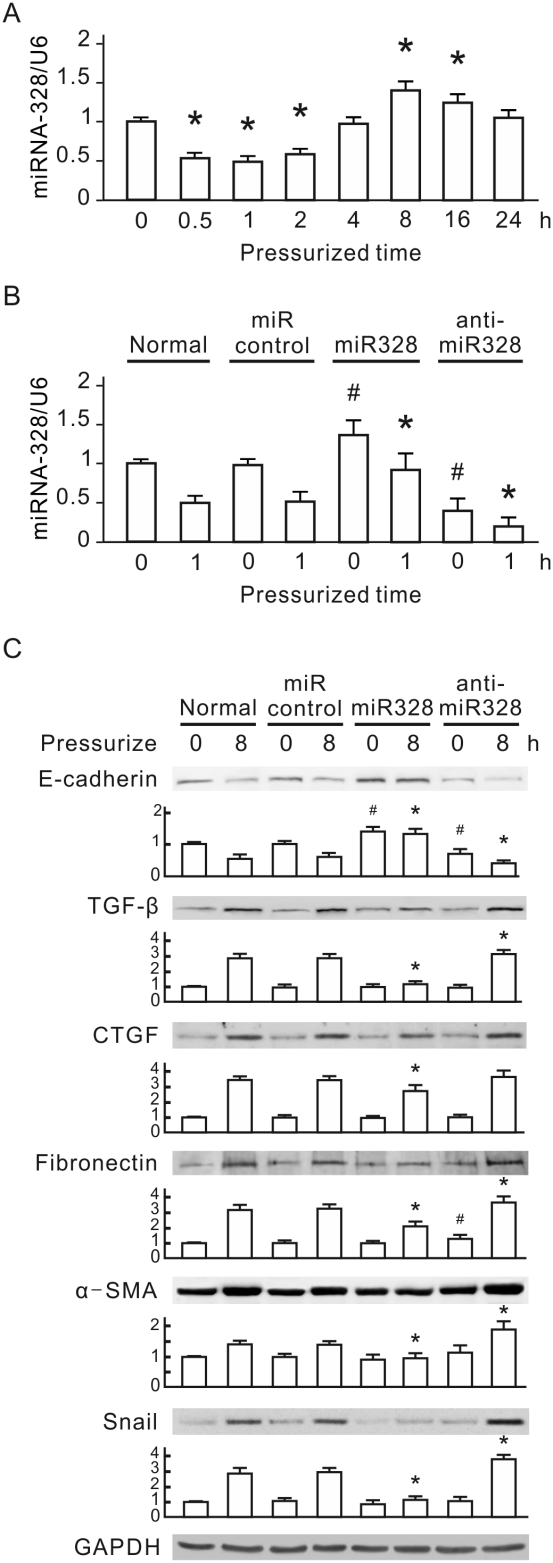
Pressure-induced miR-328 downregulation in NRK-52E cells. (A) The expression levels of miR-328 in pressurized NRK-52E cells. NRK-52E cells were pressurized for 0.5, 1, 2, 4, 8, 16 and 24 h, and the expression levels of miR-328 were analyzed by quantitative real time PCR. The relative amount of miR-328 was normalized to U6 snRNA. Triplicate assays were performed for each RNA sample. *P<0.05 vs. the group at 0 h. (B) The expression levels of miR-328 in miR-328 and miR-328 inhibitor transfected cells. NRK-52E cells were transfected with miR-328 or miR-328 inhibitor, and then pressurized for 1 h. (C) Expression of EMT markers in miR-328 and miR-328 inhibitor transfected cells. Cellular E-cadherin, TGF-β, CTGF, fibronectin, α-SMA and Snail were detected by Western blotting. GAPDH was detected as the loading control. Relative increases in the protein bands are also presented in bar chart form. Results are expressed as the mean±s.d. (n = 3). Normal, normal cells without any tranfection. miR control, control miRNA transfection. miR328, miR-328 transfection. anti-miR328, miR-328 inhibitor transfection. #*P*<0.05 vs. the miRNA control group at 0 h. *P<0.05 vs. the miRNA control group at 1 h. EMT, epithelial-mesenchymal transition; TGF-β, transforming growth factor-β; CTGF, connective tissue growth factor; α-SMA, alpha smooth muscle actin; GAPDH, glyceraldehyde-3-phosphate dehydrogenase.

We investigated the expression of miRNAs under pressure at an early stage, performing array-based miRNA profiling of NRK-52E cells under 60 mmHg pressure for 0 or 1 h. Microarray analysis of 387 rat miRNAs showed that 4 miRNAs were upregulated and 11 miRNAs were downregulated after 1 h of pressurization (Table 1). MiR-328 showed a different expression and was further characterized. We validated the miR-328 expression by qRT-PCR. MiR-328 was shown to be transiently downregulated under 60 mmHg pressure; the decrease started at 30 min and reached the lowest level at 1 h, persisting through 2 h (Fig. 2A).

**Table 1:** Summary of pressure-regulated microRNAs in NRK-52E cells.

microRNA	Expressiontrends	Quantitative index at 0 h	Quantitative index at 1 h	Expression ratio (1/0 h)
**rno-miR-193-5p**	up	0.10	4.09	40.91
**rno-miR-743a**	up	7.13	17.07	2.39
**rno-miR-878**	up	7.31	16.55	2.27
**rno-miR-223-3p**	up	6.45	13.04	2.02
**rno-miR-328**	down	488.72	272.08	0.56
**rno-miR-346**	down	446.90	233.17	0.52
**rno-let-7d-3p**	down	47.36	18.31	0.39
**rno-miR-207**	down	49.02	10.05	0.20
**rno-miR-301b-3p**	down	3.23	0.10	0.03
**rno-miR-349**	down	4.62	0.10	0.02
**rno-miR-129-5p**	down	6.48	0.10	0.02
**rno-miR-322-3p**	down	6.75	0.10	0.01
**rno-miR-378a-5p**	down	15.28	0.10	<0.01
**rno-miR-204-5p**	down	19.40	0.10	<0.01
**rno-miR-211-5p**	down	23.48	0.10	<0.01

### miR-328 Mediated EMT

To investigate whether miR-328 plays a role in pressure-induced EMT, we transfected the miR-328 precursor, the miR-328 inhibitor or miRNA control into NRK-52E cells and processed under 60 mmHg pressure. Compared to control cells, miR-328 levels significantly increased in miR-328 precursor-transfected cells and decreased in miR-328 inhibitor-transfected cells (Fig. 2B). To investigate the influence of miR-328 expression pressure-induced EMT, the expression of E-cadherin, TGF-β, CTGF, fibronectin, α-SMA and Snail was detected by Western blot. In miR-328 precursor-transfected cells, the basal level of E-cadherin significantly increased (Fig. 2C). After 8 h of pressurization, transfection with the miR-328 precursor prevented the pressure-induced decrease in E-cadherin, and significantly reduced TGF-β, CTGF, fibronectin, α-SMA and Snail as compared with control cells. In miR-328 inhibitor-transfected cells, the basal level of E-cadherin significantly decreased and that of fibronectin increased. After 8 h of pressurization, E-cadherin was further reduced, and the levels of TGF-β, fibronectin, α-SMA and Snail were significantly induced as compared with control cells. But transfection with the miR-328 inhibitor did not influence the pressure-induced expression of CTGF (Fig. 2C).

### CD44 Induced by Pressure, Downregulated by miR-328

**Figure 3:**
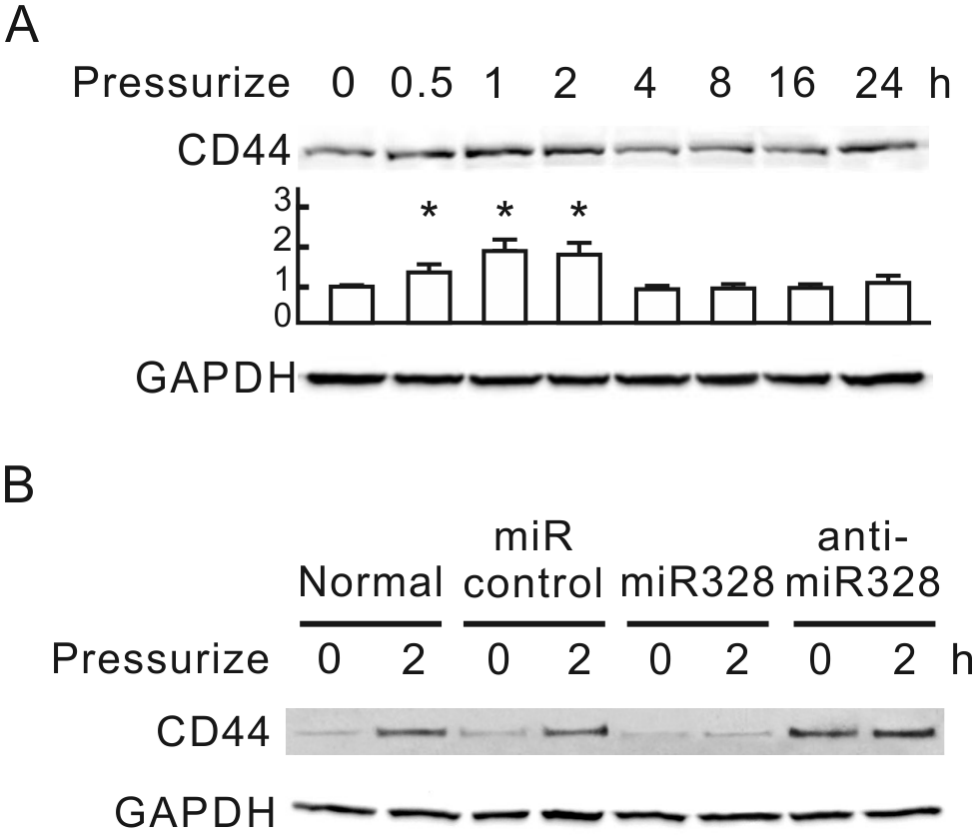
Expression of CD44 induced by a pressure of 60 mmHg in NRK-52E cells. (A) The expression levels of CD44 in pressurized NRK-52E cells. NRK-52E cells were subjected to 60 mmHg of pressure for the indicated periods. Cellular CD44 was detected by Western blotting. GAPDH was detected as the loading control. Relative increases in the protein bands are also presented in bar chart form. Results are expressed as the mean±s.d. (n = 3). *P<0.05 vs. the group at 0 h. (B) The expression levels of CD44 in miR-328 and miR-328 inhibitor transfected cells. NRK-52E cells were transfected with miR-328 or miR-328 inhibitor and then pressurized for 2 h. Cellular CD44 was detected by Western blotting. GAPDH was detected as the loading control. GAPDH, glyceraldehyde-3-phosphate dehydrogenase.

CD44 has been shown to be a potential target of miR-328 in human cells, but the miR-328-targeting sequences found in 3′-UTR of human CD44 do not exist in 3′-UTR of rat CD44. To reveal the relationship between miR-328 and CD44, we detected the expression of CD44 protein with pressurization. Fig. 3A shows that the expression of CD44 in NRK-52E cells transiently increased after 30 min under 60 mmHg pressure, peaked at 1 h, and persisted through 2 h. The time-dependent changes of CD44 protein levels were inversely correlated with miR-328 under 60 mmHg pressure from 0 to 4 h. To further determine whether miR-328 negatively regulates CD44 expression in NRK-52E cells with pressurization, we transfected NRK-52E cells with the miR-328 precursor, the miR-328 inhibitor or control miRNAs, and detected CD44 by Western blot. After 2 h of pressurization, the miR-328 precursor dramatically inhibited the pressure-induced expression of CD44 (Fig. 3B). The transfection of miR-328 inhibitor dramatically increased the basal level of CD44 and promoted the pressure-induced expression of CD44. These results suggest that miR-328 downregulates CD44 expression in NRK-52E cells.

### miR-328 Directly Targets CD44

**Figure 4:**
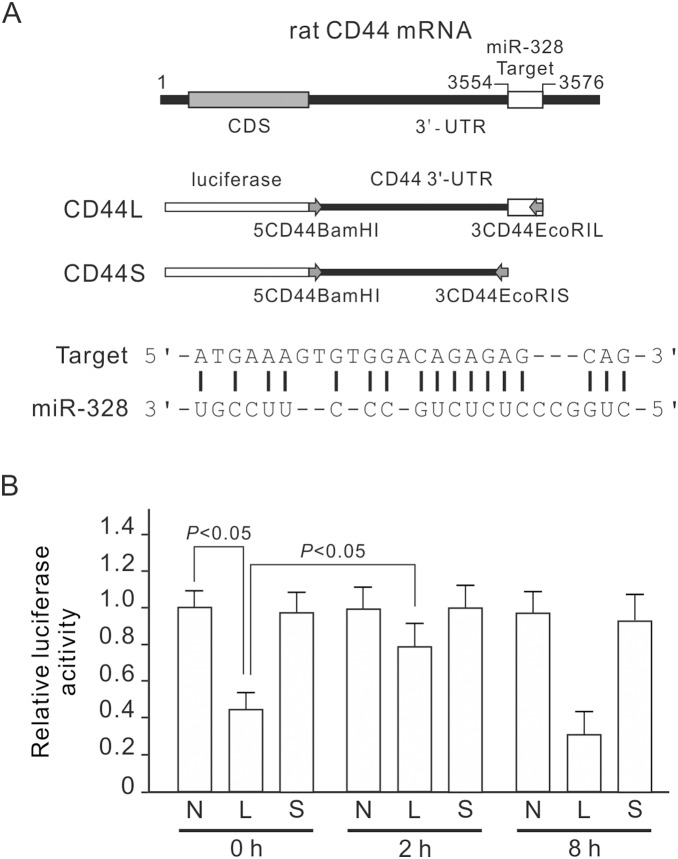
Confirmation of miR-328 targeting CD44. (A) A potential miR-328 target sequence in rat CD44 3′-UTR and luciferase reporter construct design. (B) The influence of CD44 3′-UTR on luciferase assay. NRK-52E cells were transfected with luciferase reporter constructs, which has been engineered with different fragments of the CD44 3′-UTR with or without the target sequence of miR-328 (CD44L and CD44S). The luciferase reporter lentivector alone was used as a negative control. The stable clones were then subjected to 60 mmHg of pressure for the indicated periods. Luciferase activity of CD44L-transfected cells was significantly reduced at 0 h, and significantly increased at 2 h. Relative luciferase activities are expressed as the mean±s.d. (n = 3). N, negative control; L, CD44L construct; S, CD44S construct; CDS, coding region sequence; 3′-UTR, 3′-untranslated region.

We then used the basic local alignment search tool (BLAST) to analyze antisense matches of rat CD44 3′-UTR (GeneBank access number NM_012924.2) against miR-328 and found a highly complementary sequence of miR-328 (Fig. 4A). To reveal direct repression of CD44 expression by miR-328, we integrated fragments of the CD44 3′-UTR with the complementary sequence of miR-328 into a luciferase report vector. Without pressure treatment, luciferase activity was significantly repressed in the construct containing the complementary sequence (CD44L), as compared with the control vector without the complementary sequence (CD44S, Fig. 4B). After 2 h of pressurization, luciferase activity was recovered in the CD44L construct, but pressurization for 8 h significantly decreased luciferase activity of the CD44L construct. The luciferase activity expression pattern of the CD44L construct was inversely correlated with miR-328 expression. Pressure did not influence luciferase activity of the CD44S construct. This result suggests that the highly complementary sequence of miR-328 in CD44 3′-UTR (5′-atgaaagtgtggacagagagcag) is a target of miR-328 and essential for miR-328 repression of CD44 expression.

### CD44 siRNA Prevents Pressure-induced EMT

**Figure 5:**
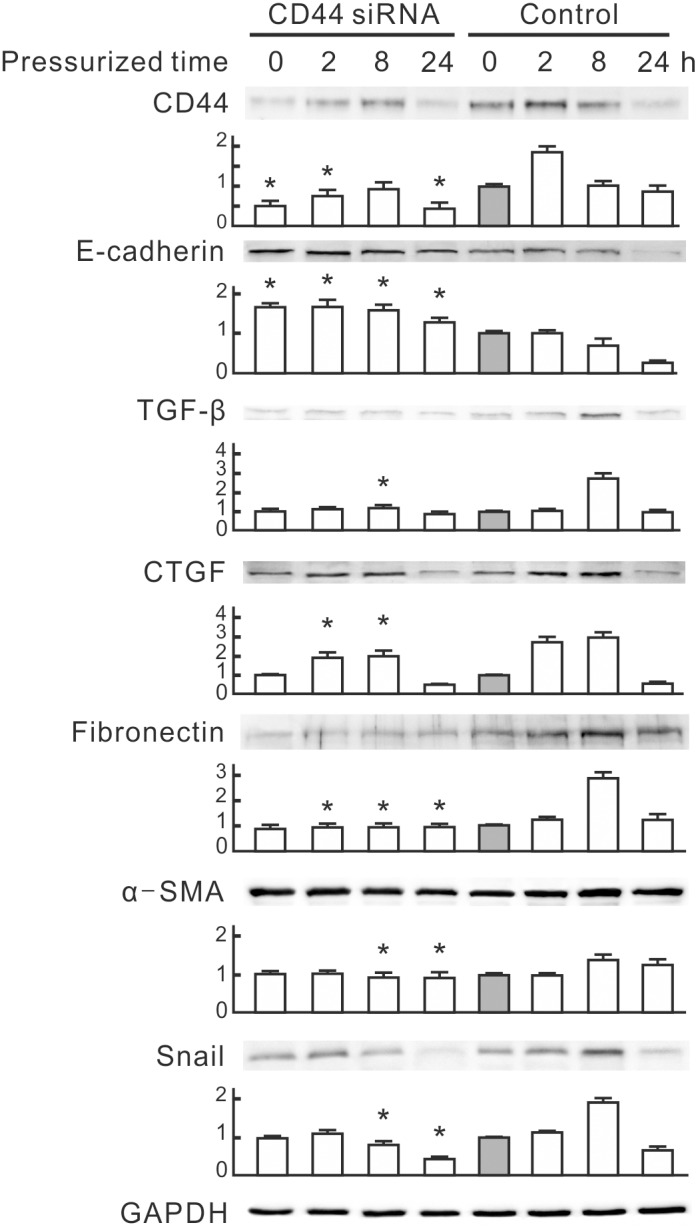
The influence of CD44 siRNA transfection on pressure-induced EMT markers. NRK-52E cells were transfected with CD44 siRNA or control siRNA, and then subjected to 60 mmHg of pressure for the indicated periods. Cellular CD44, E-cadherin, TGF-β, CTGF, fibronectin, α-SMA and Snail were detected by Western blotting. GAPDH was detected as the loading control. Relative increases in the protein bands are also presented in a bar chart form. Results are expressed as the mean±s.d. (n = 3). *P<0.05 vs. the control group at the same time point. EMT, epithelial-mesenchymal transition; TGF-β, transforming growth factor-β; CTGF, connective tissue growth factor; α-SMA, alpha smooth muscle actin; GAPDH, glyceraldehyde-3-phosphate dehydrogenase.

To investigate the influence of CD44 on pressure-induced EMT, CD44 siRNA or scrambled siRNA was transfected into NRK-52E cells, pressurized for 0, 2, 8 and 24 h. CD44 siRNA transfection significantly reduced CD44 expression levels at 0, 2, and 24 h (approximately 50–70%) as compared with scrambled siRNA transfection (Fig. 5). The scrambled siRNA transfected cells developed an EMT phenotype during pressure treatment accompanied by downregulation of E-cadherin and upregulation of TGF-β, CTGF, fibronectin, α-SMA and Snail. CD44 siRNA transfection significantly increased the basal level of E-cadherin and prevented pressure-induced decreases of E-cadherin. NRK-52E cells transfected with CD44 siRNA demonstrated a significant reduction of pressure-induced TGF-β, CTGF, fibronectin, α-SMA and Snail compared with negative controls (Fig. 5), indicating that CD44 plays a critical role in pressure EMT in NRK-52E cells.

### Blockage of HA and OPN Reduces Pressure-induced EMT

**Figure 6:**
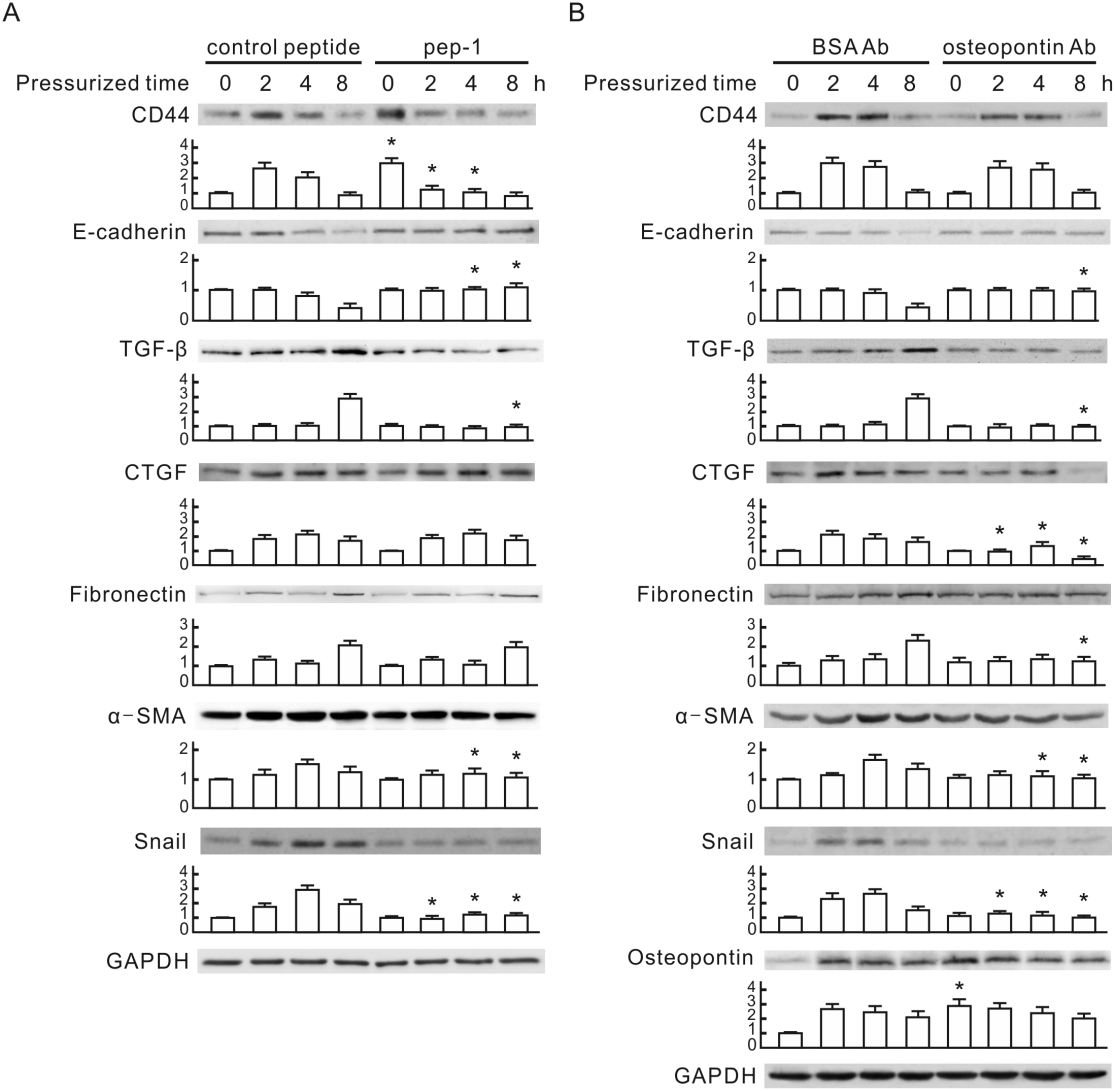
The involvement of hyaluronan and osteopontin in pressure-induced EMT. (**A**) The influence of pep-1 on pressure-induced EMT markers. NRK-52E cells were pretreated with pep-1 (100 µg/ml) or the control peptide for 30 min, and then subjected to 60 mmHg of pressure for the indicated periods. (**B**) The influence of osteopontin neutralizing antibody on pressure-induced EMT markers. NRK-52E cells were pretreated with or without osteopontin neutralizing antibody (1 µg/ml) or control BSA antibody for 30 min, and then subjected to 60 mmHg of pressure for the indicated periods. GAPDH was detected as the loading control. Relative increases in the protein bands are also presented in a bar chart form. Results are expressed as the mean±s.d. (n = 3). *P<0.05 vs. the control group at the same time point. EMT, epithelial-mesenchymal transition; TGF-β, transforming growth factor-β; CTGF, connective tissue growth factor; α-SMA, alpha smooth muscle actin; BSA, bovine serum albumin; GAPDH, glyceraldehyde-3-phosphate dehydrogenase.

We investigated whether the main ligands of CD44, HA and OPN, were necessary for pressure-induced EMT in tubular epithelial cells. NRK-52E cells were treated with an HA binding peptide (Pep-1,500 µg/ml) to displace pericellular HA, and demonstrated a significant reduction on pressure-induced TGF-β, α-SMA and Snail compared with negative controls (Fig. 6A). Pep-1 also prevented a pressure-induced decrease of E-cadherin, but did not influence the expression of CTGF and fibronectin in NRK-52E cells treated with pressure. Pep-1 treatment increased the basal level of CD44 and decreased pressure-induced CD44 (Fig. 6A). We used OPN neutralizing antibody to neutralize secreted OPN in culture medium and found that OPN neutralizing antibody treatment prevented a pressure-induced decrease of E-cadherin and reduced the pressure-induced increase of TGF-β, α-SMA and Snail compared with BSA antibody controls (Fig. 6B). OPN neutralization further reduced the expression of CTGF and fibronectin in NRK-52E cells treated with pressure. OPN neutralizing antibody did not influence the basal level of CD44 and inhibited CD44 expression in pressure-treated cells (Fig. 6B). Additionally, the pressure of 60 mmHg induced OPN expression at 2, 4 and 8 h. OPN neutralization significantly increased the basal level of OPN in NRK-52E cells but did not influence the pressure-induced increase of OPN (Fig. 6B). These results reveal that the interaction between CD44 and its ligands is involved in pressure-induced EMT.

### miR-328 and CD44 Expression in UUO Rat Renal Tissues

**Figure 7:**
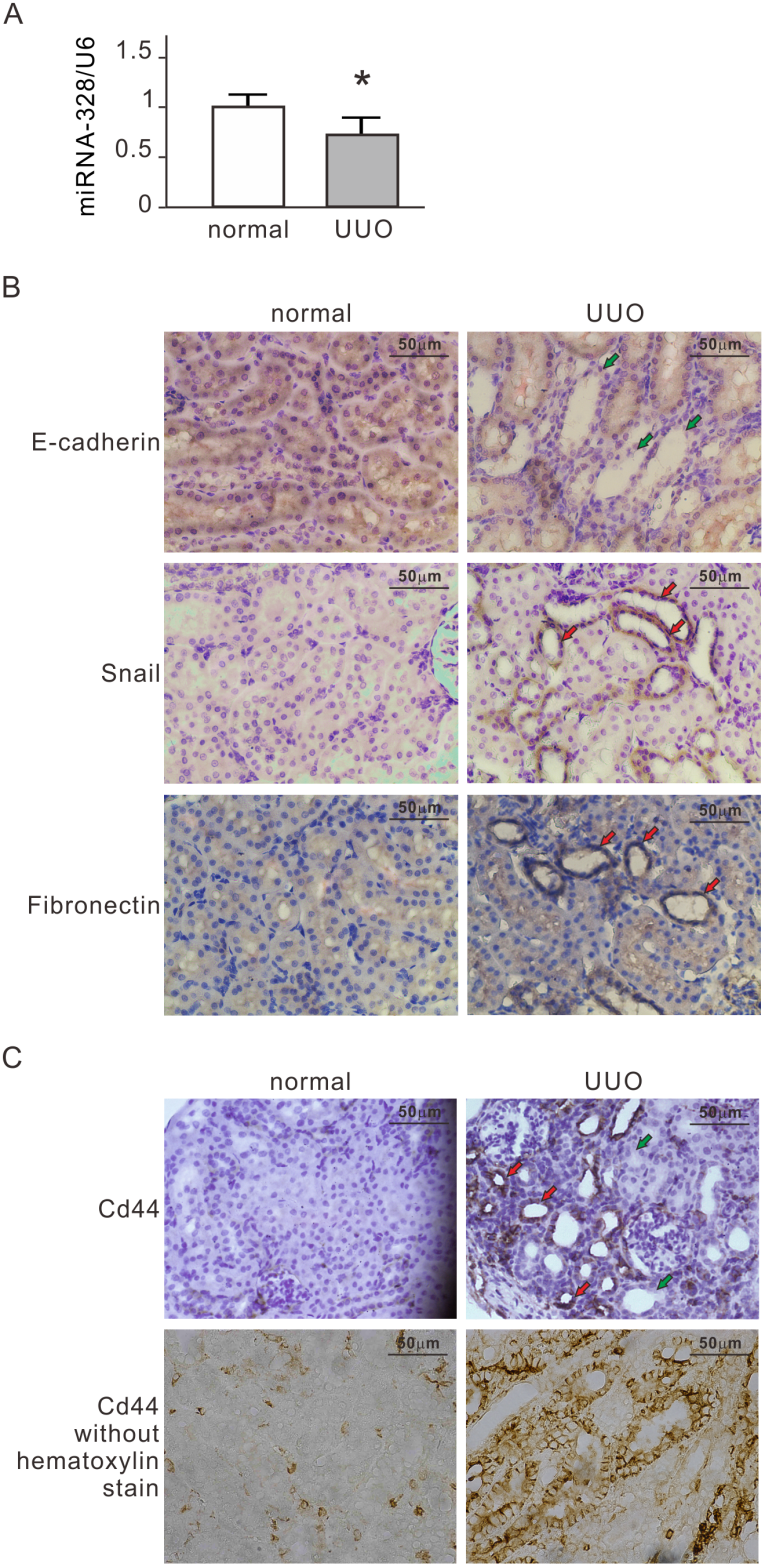
Expression of miR-328 in renal tissue from rats with unilateral ureteral obstruction (UUO). (A) Expression analysis of miR-328 in renal tissue from UUO rats by quantitative real time PCR. The relative expression values of miR-328 were normalized to U6. The normal kidneys in UUO rats were used as control. Results are expressed as the mean±s.d. (n = 5). *P<0.05. (B) Expression of E-cadherin, Snail and fibronectin in renal tissue from UUO rats by immunohistochemistry. The normal kidneys in UUO rats were used as control. The green arrow indicates the negative label of E-cadherin. The red arrow indicates the positive label of Snail or fibronectin. (C) Expression of CD44 in renal tissue from UUO rats by immunohistochemistry. Strong membranous labeling of CD44 was observed in renal obstruction kidneys as revealed by immunohistochemistry without hematoxylin stain. The green arrow and red arrow indicate the negative label and the positive label of CD44 respectively. UUO, unilateral ureteral obstruction.

We monitored the expression of miR-328 in tubular epithelial cells in renal tissues of UUO rats. Quantitative RT-PCR analyses revealed reduced miR-328 levels in UUO renal tissues as compared with normal renal tissues (Fig. 7A, *P*<0.05). We then detected the epithelial marker E-cadherin and mesenchymal markers Snail and fibronectin in UUO renal tissues with immunohistochemistry (IHC). Renal obstruction causes tubular atrophy in UUO renal tissues (Fig. 7B). Positive staining of E-cadherin and weak signals of Snail and fibronectin were observed in normal tubular epithelial cells, whereas reduced labeling of E-cadherin and strong staining of Snail and fibronectin were observed in the tubular epithelial cells of dilated renal tubules (Fig. 7B). These results demonstrated that tubular epithelial cells of UUO kidneys are developing the mesenchymal phenotype. IHC stain also revealed that strong membranous labeling of CD44 was observed in tubular cells in partially dilated renal tubules but not in normal renal tubules of UUO kidneys (Fig. 7C). These data further support the findings that miR-328 and CD44 are involved in renal EMT as observed in NRK-52E cells.

## Discussion

**Figure 8:**
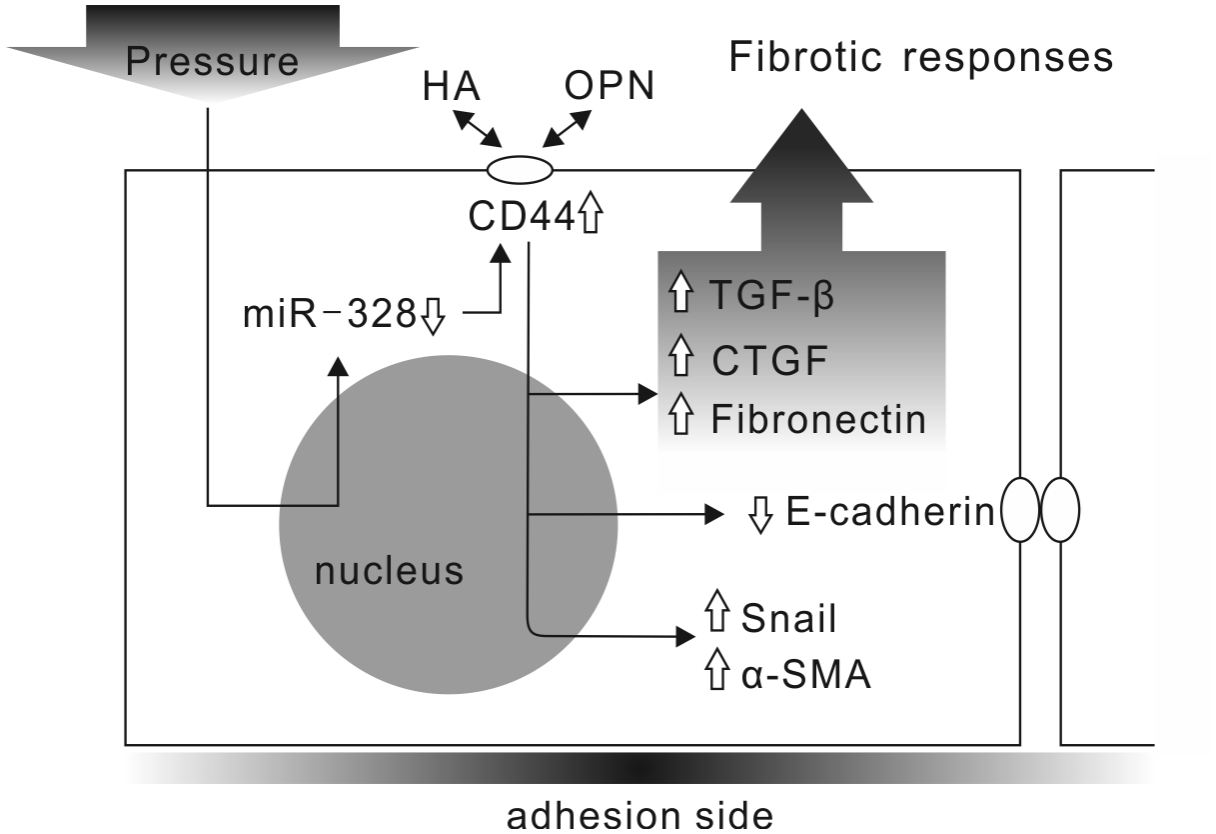
Schematic representation of miR-328/CD44-mediated epithelial-to-mesenchymal transition in renal tubular cells. The pressure transiently decreases miR-328, and then increases CD44 expression in renal tubular cells. Increased CD44 enhances the interaction between CD44 and hyaluronan (HA)/osteopontin (OPN), and further reduces epithelial marker E-cadherin and induces connective tissue growth factor (CTGF), transforming growth factor (TGF)-β, and mesenchymal markers, such as alpha smooth muscle actin (α-SMA), fibronectin and Snail. CTGF and TGF-β are important paracrine factors to induce EMT and fibrosis in renal tubules.

This study used a pressurized cell culture system to investigate pressure-induced EMT in NRK-52E cells. The pressure of 60 mmHg induced the expression of CTGF and TGF-β, decreased epithelial marker E-cadherin, and increased mesenchymal markers, such as α-SMA, fibronectin and Snail, in NRK-52E cells (Fig. 1). MicroRNA array assays showed that pressure reduced miR-328 at the initial stage of pressurization. The expression level of miR-328 in pressurized cells significantly decreased at 1–2 h and influenced pressure-induced EMT (Fig. 2). The expression pattern and 3′-UTR analysis revealed that CD44 was the target of miR-328 in pressurized NRK-52E cells (Fig. 3 and 4). The transfection of CD44 siRNA confirmed the promoting role of CD44 in pressure-induced EMT (Fig. 5). We also found that miR328 expression in UUO kidneys was less than that in normal kidneys, and CD44 increased in renal tubular cells in slightly dilated tubules (Fig. 7). Both HA binding peptide pep-1 and OPN neutralizing antibody inhibited pressure-induced EMT (Fig. 6). The interaction between CD44 and its ligands is necessary for pressure-induced EMT, and induced CD44 increases HA- and OPN-mediated signals. Here, we presented the first data showing that the transient downregulation of miR-328 by pressure could increase CD44 to induce renal tubular cell EMT, a major contributor to the pathogenesis of renal fibrosis, in tubular epithelial cells (Fig. 8). CD44 and miR-328 may serve as biomarkers and potential therapeutic targets for pressure-associated renal fibrosis.

Pressure is thought to be a major mechanism to induce renal fibrosis in ureteric obstruction. Recent evidence suggests that the pressure force also contributes to the induction and progression of tubulointerstitial fibrogenesis in diabetic nephropathy [26]. Understanding the early process of pressure-induced renal fibrosis should be beneficial for treatment of chronic kidney diseases. EMT is an early key mechanism in the pathogenesis and progression of renal interstitial fibrosis [9]. Blocking the tubular EMT inhibits renal interstitial fibrosis in UUO rats [27]. Our study provides in vitro evidence that pressure induces renal tubular EMT at an early stage. We suggest that pressure-induced EMT plays a critical role in progression of renal interstitial fibrosis in ureteric obstruction. TGF-β is thought to be an important mediator in EMT and renal fibrosis [12], [28]. In our system the time point of TGF-β induction in pressurized NRK-52E cells is later than those of miR-328 reduction and CD44 induction (Fig. 1 and 3). Both overexpression of miR-328 and knockdown of CD44 inhibited the pressure-induced TGF-β and EMT markers (Fig. 2 and 5). These results suggest that miR-328 and CD44 are upstream regulators of TGF-β, and miR-328-mediated CD44 transient upregulation is an important trigger of pressure-induced EMT.

EMT is proposed as a mechanism of many organs, such as kidney, liver, heart and lung [29]–[32]. But some researchers doubt about the existence of EMT in renal fibrosis [33], [34]. Their lineage tracing studies do not support a substantial contribution of EMT to nephron loss, epithelial origin of myofibroblasts and renal interstitial fibrosis in UUO mice. A recent clinical study with 23 patients showed that tubular epithelial cells in human fibrotic kidneys decreased expression of epithelial biomarkers and increased expression of mesenchymal biomarkers [35]. This finding reveals a significant correlation between EMT and renal fibrosis in human. Blocking the renal tubular EMT by norcantharidin inhibits renal interstitial fibrosis in UUO rats [27]. Our results also show that tubular epithelial cells in UUO rat kidneys decrease E-cadherin expression and increase fibronectin and Snail expression. Therefore, the existence of EMT in renal fibrosis is believable.

Many studies show that miR-328 is expressed at low levels in many cancers, and acts as a tumor suppressor. MiR-328 expression was decreased in high-grade gliomas and was associated with poor survival in primary glioblastoma patients [36]. In colorectal cancer, the expression of miR-328 was downregulated in side population cells [37]. The over-expression of miR-328 inhibited cell invasion of side population cells. MiR-328 also influenced drug efflux by repressing breast cancer resistance protein mRNA expression in breast cancer cells [38]. Expression of miR-328 reduced cell adhesion, aggregation, and migration in a human epidermal carcinoma cell line [20]. The direct effects of miR-328 have been studied in a wide range of cancer cells. Relatively few studies regarding miR-328 in other cellular functions have been reported. We demonstrated for the first time that miR-328 plays a role in renal fibrosis in ureteric obstruction. Our data showed that miR-328 over-expression suppressed pressure-induced tubular epithelial cell EMT. EMT is thought to be involved in the induction of cellular traits associated with the metastatic progression of cancers [39]. The inhibitory effect of miR-328 on EMT also supports its role as a tumor suppressor.

CD44 is one the proteins downregulated by miR-328 in human cells [20], and its expression is reported to be associated with EMT in nasopharyngeal carcinoma [40]. Wu et al. reported that UUO in mice resulted in significantly increased RNA expression of CD44 postoperatively [41]. These results inspired us to study the role of CD44 in pressure-induced EMT. In human cells, miR-328 has been reported to target the 3′-UTR of CD44 mRNA [38]. The miR-328 target sequence in human CD44 3′-UTR does not exist in rat CD44 3′-UTR. Three microRNA target prediction websites, TargetScan (www.targetscan.org), microRNA.org (www.microrna.org) and miRBase (www.mirbase.org), suggest no potential miR-328 target sequence in rat CD44 3′-UTR. We used the BLAST to screen antisense matches of rat CD44 3′-UTR against miR-328, and then found a potential target sequence (Fig. 4A). This potential target sequence was further proved to be the miR-328 target sequence in rat CD44 3′-UTR (Fig. 4B). Our study provides a new miR-328 target sequence in rat CD44 3′-UTR by which miR-328 negatively regulates the expression of CD44 in rat renal tubular cells.

CD44 is a cell surface glycoprotein involved in cell proliferation, cell differentiation, cell migration, angiogenesis, and cell survival [42]. These biological properties are essential to the physiological activities of normal cells but are also associated with the pathologic progression of fibrosis and cancer. In nasopharyngeal carcinoma, CD44 plays a role in the EMT phenotype of cancer stem-like cells [40]. Treatment with a CD44-blocking antibody is shown to reduce lung fibrosis in mice [43]. CD44 is the major receptor of HA and OPN [22], [23]. HA, a water-soluble glycosaminoglycan, is a key component of the pericellular matrix and has important structural functions in the extracellular matrix of all tissues. Over-expression of HA synthase 2 cDNA increased HA synthesis and the pericellular HA matrix with a corresponding increase in renal tubular cell migration [44]. Increased HA also produced an aggressive phenotype leading to severe lung fibrosis and death after bleomycin-induced injury [43]. OPN is a phosphorylated glycoprotein that exists as an immobilized matrix protein and a cytokine [45]. Recent evidence suggests that OPN plays a key role in fibrosis [46], [47]. Reduction of OPN by siRNA transfection in vivo inhibited progression of chronic allograft nephropathy [48]. Our study used pep-1 and OPN neutralizing antibody to interrupt the CD44 and HA/OPN interaction and found both interruptions prevented the pressure-induced decrease of E-cadherin and also reduced the pressure-induced increase of TGF-β, α-SMA and Snail (Fig. 6). OPN neutralization further decreased the pressure-induced expression of CTGF and fibronectin in NRK-52E cells (Fig. 6B). These results show that the CD44 and HA/OPN interaction is involved in pressure-induced renal tubular cell EMT. Since EMT may be reversed with timely removal of the surrounding pathogenic factors [49], our finding help explain this phenomenon.

In summary, we present the first evidence that miRNAs are involved in the development of pressure-induced EMT in tubular epithelial cells. Pressure-mediated transient downregulation of miR-328 promotes EMT in tubular epithelial cells by upregulating CD44. CD44 and HA/OPN interaction plays an important role in pressure-induced EMT in renal tubular cells. Our data clarify the mechanisms of pressure-induced EMT, establishing a rationale for developing miR-328 and CD44 as therapeutic targets for pressure-induced renal fibrosis.
